# Rare cancers of unknown etiology: lessons learned from a European multi-center case–control study

**DOI:** 10.1007/s10654-020-00663-y

**Published:** 2020-07-17

**Authors:** Elsebeth Lynge, Linda Kaerlev, Jørn Olsen, Svend Sabroe, Noemia Afonso, Wolfgang Ahrens, Mikael Eriksson, Franco Merletti, Maria Morales-Suarez-Varelas, Aivars Stengrevics, Pascal Guénel

**Affiliations:** 1 Nykøbing Falster Hospital, University of Copenhagen, Ejegodvej 63, 4800 Nykøbing Falster, Denmark; 2 Research Unit of Clinical Epidemiology, Department of Clinical Research, University of Southern Denmark, Odense, Denmark; 3 Center for Clinical Epidemiology, Odense University Hospital, Kløvervænget 30, 5000 Odense C, Denmark; 4 Department of Clinical Epidemiology, Aarhus University Hospital, Palle Juul-Jensens Boulevard 99, 8200 Aarhus N, Denmark; 5 Department of Public Health, Aarhus University, Bartholins Allé 2, 8000 Aarhus C, Denmark; 6 Serviço de Oncologia, Centro Hospitalar E Universitário Do Porto, Largo Do Prof. Abel Salazar, 4099-001 Porto, Portugal; 7 Faculty of Mathematics and Computer Science, University of Bremen, Bremen, Germany; 8 Leibniz Institute for Prevention Research and Epidemiology – BIPS, Achterstrasse 30, E 28359 Bremen, Germany; 9 Skane University Hospital and Lund University, Entrégatan 7, 222 42 Lund, Sweden; 10 Cancer Epidemiology, Department of Medical Sciences, University of Turin, Via Santena 7, 10126 Torino, Italy; 11 Department of Preventive Medicine and Public Health, Food Sciences, Toxicology and Legal Medicine, School of Pharmacy, University of Valencia, Avenida Vicente Andres Estellés s/n Burjassot, S46100 Valencia, Spain; 12 Biomedical Research Consortium in Epidemiology and Public Health Network (CIBERESP), Madrid, Spain; 13 A. Deglava 152/3–120, Riga, 1021 Latvia; 14 Center for Research in Epidemiology and Population Health (CESP), Cancer and Environment team, Inserm U1018, Université Paris Sud, Université Paris Saclay, 16 avenue Paul Vaillant-Couturier, 94800 Villejuif, France

**Keywords:** Risk factor, Cancer

## Abstract

Rare cancers together constitute one fourth of cancers. As some rare cancers are caused by occupational exposures, a systematic search for further associations might contribute to future prevention. We undertook a European, multi-center case–control study of occupational risks for cancers of small intestine, bone sarcoma, uveal melanoma, mycosis fungoides, thymus, male biliary tract and breast. Incident cases aged 35–69 years and sex-and age-matched population/colon cancer controls were interviewed, including a complete list of jobs. Associations between occupational exposure and cancer were assessed with unconditional logistic regression controlled for sex, age, country, and known confounders, and reported as odds ratios (OR) with 95% confidence intervals (CI). Interviewed were 1053 cases, 2062 population, and 1084 colon cancer controls. Male biliary tract cancer was associated with exposure to oils with polychlorinated biphenyls; OR 2.8 (95% CI 1.3–5.9); male breast cancer with exposure to trichloroethylene; OR 1.9 (95% CI 1.1–3.3); bone sarcoma with job as a carpenter/joiner; OR 4.3 (95% CI 1.7–10.5); and uveal melanoma with job as a welder/sheet metal worker; OR 1.95 (95% CI 1.08–3.52); and cook; OR 2.4 (95% CI 1.4–4.3). A confirmatory study of printers enhanced suspicion of 1,2-dichloropropane as a risk for biliary tract cancer. Results contributed to evidence for classification of welding and 1,2-dichloropronane as human carcinogens. However, despite efforts across nine countries, for some cancer sites only about 100 cases were interviewed. The Rare Cancer Study illustrated both the strengths and limitations of explorative studies for identification of etiological leads.

## Introduction

Rare cancers together constitute a considerable part of the cancer burden. In Europe, a rare cancer was defined as a cancer with an incidence of less than six per 100,000. This led to identification of 198 disease entities, together constituting 24% of cancers diagnosed in the European Union [1]. Occupational exposures are known risk factors for several rare cancers; examples are asbestos and pleural mesothelioma, and benzene and acute myeloid leukemia [2].

The potentially occupational origin of rare cancers has in most cases been suggested by alert clinicians. Exposure to wood dust as a risk factor for nasal adenocarcinomas was based on a cluster in furniture-makers in Buckinghamshire, United Kingdom [3]. A causal link between vinyl chloride and liver angiosarcoma was suggested by two company physicians observing four cases among workers in the polymerization section of a plant in Kentucky, United States [4]. Recently, a cluster of cholangiocarcinomas was observed in a small offset color-proof printing facility in Osaka, Japan, where workers had been exposed to 1,2-dichloropropane and dichloromethane [5].

On this background, one might hypothesize that a systematic search for associations between occupational exposures and rare cancers would reveal new etiological leads. To obtain sufficient numbers, patients for such a study should be recruited from a large population. We undertook a European multi-center case–control study on risk factors for seven rare cancers. Incident cases and controls were recruited from nine European countries with personal interviews of 4000 participants. Here we report on selected key findings and in light of our experiences discuss strengths and limitations of this study approach.

## Material and methods

**Fig. 1:**
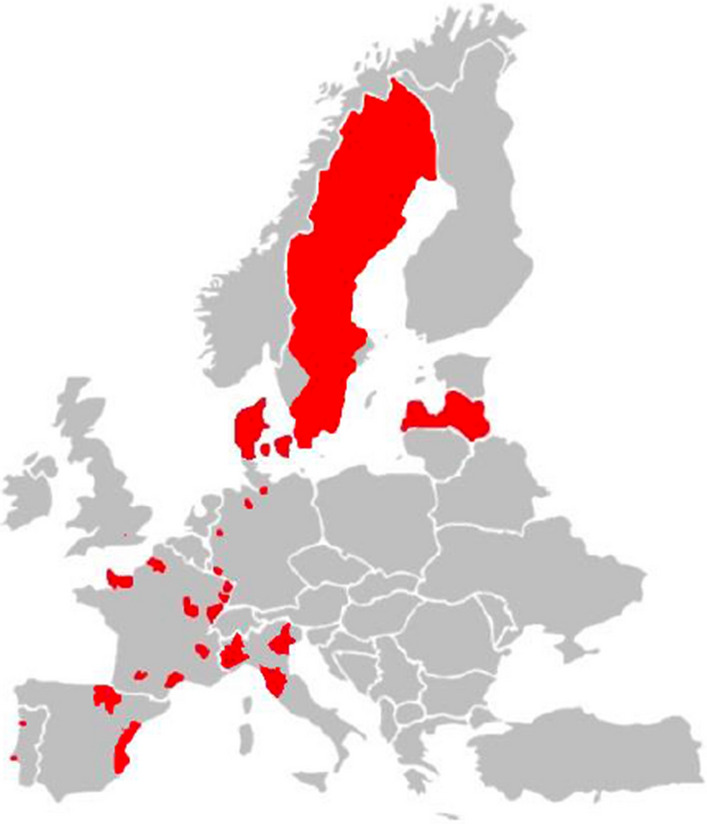
Geographical regions covered by the European multi-center case–control study on rare cancers of unknown etiology

The Rare Cancer Study aimed to serve both as a confirmatory study of specified hypotheses, and as an explorative study. The design was described previously [6]. In short, incident cases aged 35–69 years diagnosed 1995–1997 with cancers of the small intestine, bone sarcoma, uveal melanoma, mycosis fungoides, thymus, and male biliary tract and male breast were recruited. The lower age limit was set to allow for some time for accumulation of occupational exposures prior to age of diagnosis, and the upper age limit was set to avoid comorbidities that would incapacitate participation. The seven cancer sites were chosen based on a literature review [7], which indicated that occupational risk factors could be involved in the etiology of these diseases. Population-based in Denmark and Latvia, in ten areas in France, five in Germany, three in Italy, and four in Sweden; hospital-based in three places in Spain, two in Portugal, and at one eye-hospital in United Kingdom, Fig. 1. Cases were reviewed by an expert pathologist. Sex and age-matched population-controls four times the most frequent cancer were selected from Denmark, France, Germany, Italy, and Sweden, and colon cancer controls from Denmark, and from Latvia, Spain, and Portugal where population-controls could not be selected.

We aimed for interviewing all identified cases and selected controls. To ensure a high response rate, cases were interviewed as soon as possible after the diagnosis. If a case had died or was unable to participate, we aimed for interviewing a next-of-kin. Controls were interviewed in batches throughout the data collection period. The pathology review was undertaken in parallel with the interviews, and in the analysis we included only interviewed cases for whom the diagnosis was considered to be definite or possible. The core questionnaire covered demographic variables, eye color, medical and x-ray history, use of drugs, tobacco and alcohol, and occupational exposures as organic solvents, pesticides, and electromagnetic fields. A complete history was obtained of all jobs lasting at least six months including data on working hours, materials handled, and chemical exposures. In addition, using the method by Siemiatychi [8] we used 27 job-specific supplementary questionnaires providing a comprehensive picture of the exposures in given job (ref). Questionnaires were translated into the languages of the respective countries. Jobs were coded using the International Classification of Occupations from 1968 [9], and the European Classification of Industries from 1993 [10].

Associations between occupational exposure and cancer were assessed with unconditional logistic regression controlled for sex, age, country, and known confounders, and reported as odds ratios (OR) with 95% confidence intervals (CI). Selected known associations with medical conditions were studied to check data validity, before associations with occupational exposures were explored. The study was undertaken in accordance with Ethical Committee requirements in each country.

## Results

In total, 1457 patients were recruited, with diagnosis assessed as definite/possible for 1252, and 1053 were interviewed, almost 90% in-person, Table 1.

**Table 1:** European rare cancer study. number of identified and interviewed cases and controls

Cancer site/group	Identified	Definite/possible diagnosis	Interviewed^d^	Percent interviewed/identified
Uveal melanoma	359	317	289	81%
Male biliary tract	295	258	186	63%
Small intestine	251	216	171	68%
Adenocarcinoma			70^c^	
Carcinoid			84^c^	
Bone	138	116	105	76%
Thymus	137	113	101	74%
Mycosis fungoides	147	115	101	69%
Male breast	123	117	100	81%
Total	1457^a^	1252	1053	72%
Population control	3374	NR	2062	61%
Colon cancer control	1284^b^	NR	1084	86%^b^

NR Not relevant

^a^Includes 7 cases of unknown site; ^b^may indicate some incomplete identification of eligible controls; ^c^numbers from papers where the two subgroups were reported separately; ^d^due to missing values on some variables, the actual number of cases and controls included in a given analysis could be lower than the numbers interviewed. The numbers might also be slightly higher, reflecting for instance extra case recruitment of uveal melanoma cases in Germany

### Use of colon cancer controls

In Denmark, both 320 population and 254 colon cancer controls were recruited. The groups were similar in education, medical history and smoking, but colon cancer controls had higher alcohol intake, less frequent work as a farmer, and less exposure to pesticides than population controls [11]. These differences affected some findings.

The association between sunlight exposure and uveal melanoma was OR 1.91 (95% CI 1.22–2.98) with colon cancer controls; but only OR 1.24 (95% CI 0.88–1.74) with population controls; reflecting that farmers had outdoor work and low risk of colon cancer [12]. With both population and colon cancer controls exposure to pesticides showed an excess risk with bone sarcoma; OR 2.33 (95% CI 1.31–4.13), that decreased with population controls only; OR 1.63 (95% CI 0.77–3.45) [13].

### Biliary tract carcinoma in men

Biliary tract carcinoma was studied including 153 cases and 1421 population controls. A history of gallstones is a known risk factor, and this was confirmed; OR 4.68 (95% CI 2.80–7.84) [14]. Questionnaire data on chemicals were used to construct a cumulative exposure index taking probability, intensity and duration in each job into account. In the analysis, the exposed participants were categorized in tertiles; low, medium, high, of the joint distribution of cases and controls [15]. As an example, 6%; 12%, 19% and 63% of biliary tract carcinoma cases were categorized as low, medium, high and unexposed, respectively, to endocrine-disrupting compounds. Exposure to endocrine-disrupting compounds as a risk factor for male biliary tract carcinoma was studied, because the preponderance of this disease in women is assumed related to female sex hormones. The data showed an OR of 1.4 (95% CI 0.9–2.0) based on all data, and of 1.7 (95% CI 1.1–2.8) based on job-specific questionnaires, Table 2, with no dose–response relationship [15].

**Table 2:** Selected excess risks of occupational exposures for rare cancers observed in the European Rare Cancer Study

References	Cancer site	Type of con-trols	Number of exposed		Number of unexposed		OR^a^	95% CI
			Cases	Con-trols	Cases	Con-trols		
[15]	Male biliary tract carcinoma							
	All endocrine disrupting compounds	P	41	477	37	716	1.7	1.1–2.8
	Oils with PCB	P	8	51	115	1820	3.2	1.4–7.4
[17]	Male breast cancer							
	Health care and social work	P	8	93	96	1808	2.3	1.1–5.1
	Forestry and logging	P	7	39	97	1862	2.4	1.0–5.6
	Wood preparation and paper makers	P	5	26	99	1875	2.4	0.9–6.5
	Motor vehicle mechanics	P	9	74	95	1827	2.1	1.0–4.4
	Painters	P	7	54	97	1847	2.3	1.0–5.2
	Alkylphenolic compounds, high	P	7	26	97	1875	3.3	1.1–9.9
[18]	Trichloroethylene, high	P + C	29	307	75	1594	1.9	1.1–3.3
[20]	Small intestine adenocarcinoma							
	Men: Manufacture of motor vehicles	P + C	4	57	40	1755	3.9	1.3–11.9
	Men: Building caretaker	P + C	4	28	40	1784	5.7	1.8–18.3
	Women: Mixed farming	P + C	7	42	28	795	3.2	1.3–8.3
	Women: Manufacture of work wear and clothes	P + C	8	68	27	769	3.5	1.5–8.3
	Women: Restaurant	P + C	6	46	29	791	2.8	1.1–7.6
	Women: Dry cleaner/launderer	P + C	4	31	31	806	3.8	1.2–12.6
	Women: General farm laborer	P + C	7	35	28	802	4.6	1.8–12.0
[21]	Small intestine carcinoid							
	Men: Manufacture of bodies for motor vehicles	P	3	11	48	1436	5.1	1.2–22.1
	Men: Structural metal preparer/erector	P	3	46	48	1401	4.3	1.2–15.9
	Men: Other construction workers	P	5	76	46	1371	3.1	1.1–8.6
	Women: Wholesale, food and beverages	P	3	13	30	610	8.2	1.9–34.9
[13]	Bone sarcoma							
	Blacksmith, toolmaker, machine-tool operator	P + C	11	199	85	2433	2.14	1.08–4.26
	Bricklayer, carpenter, other construction	P + C	14	215	82	2417	2.93	1.55–5.53
	Of whom: carpenter, joiner, parquetry	P + C	6	61	90	2571	4.25	1.71–10.5
	Manufacture of wood, wood and cork products, straw and plaiting industry	P + C	9	88	87	2544	3.58	1.70–7.56
[12]	Uveal melanoma							
	Cook	P	18	62	274	2000	2.40	1.35–4.28
	Welders and sheet metal workers	P	16	86	276	1976	1.95	1.08–3.52
	Service workers NOS	P	66	395	226	1667	1.43	1.02–2.00
	Launderers, dry-cleaners and pressers	P	10	23	282	2039	3.14	1.44–6.86

OR: odds ratio; CI: confidence interval; P: population control; C: colon cancer control; PCB: polychlorinated biphenyls; NOS: not otherwise specified

^a^Originally reported ORs with one or two digits, respectively, have been used

For the subgroup of endocrine disrupting compounds including oils with polychlorinated biphenyls (PCB) the OR was 2.8 (95% CI 1.3–5.9) for all data, and OR 3.2 (95% CI 1.4–7.4) for job-specific questionnaires; no dose–response relationship. A possible causal association for PCB was supported by an OR of 2.3 (95% CI 1.2–4.5) for men employed in electrical work, as 70% of electrical workers were classified as exposed to endocrine-disrupting compounds, including PCB [15]. No association was found with exposure to pesticides in general, and power was insufficient to distinguish between types [16].

### Male breast cancer

The study included 104 cases and 1901 population/colon cancer controls showing the known association with gynecomastia; OR 23.42 (95% CI 4.65–117.97) [17]. Excess risks were indicated in health care and social work; OR 2.3 (95% CI 1.1–5.1); forestry and logging; OR 2.4 (95% CI 1.0–5.6); wood preparation and paper makers; OR 2.4 (95% CI 0.9–6.5); motor vehicle mechanics; OR 2.1 (95% CI 1.0–4.4); painters; OR 2.3 (95% CI 1.0–5.2); and for alkylphenolic compounds used as detergents in many industries; OR 3.3 (95% CI 1.1–9.9).

Exposure to organic solvents was assessed using a cumulative exposure score constructed from the job history combined with a French Job Exposure Matrix (JEM). Exposure probability, frequency, intensity and duration in each job were taken into account. In the analysis, exposed workers were dichotomized into low and high according to the median score among the exposed controls [18]*.* For trichloroethylene this resulted in 16% of cases being categorized as low exposed; 28% as high exposed; and 56% as unexposed. The risk of male breast cancer was increased for trichloroethylene; OR 1.4 (95% CI 0.7–2.5) for low, and OR 1.9 (95% CI 1.1–3.3) for high score [18]. Results by occupation and JEM supported each other, as motor vehicle mechanics and painters were exposed to trichloroethylene.

### Small-bowel cancer

The association between Crohn’s Disease and small-bowel adenocarcinoma was confirmed; OR 53.6 (95% CI 6.0–477) [19]. Including 79 cases and 2649 population/colon cancer controls; for men increased risks were found for manufacture of motor vehicles; OR 3.9 (95% CI 1.3–11.9); and for building caretaker; OR 5.7 (95% CI 1.8–18.3). For women increased risks were found for mixed farming; OR 3.2 (95% CI 1.3–8.3); manufacture of work wear and clothes; OR 3.5 (95% CI 1.5–8.3); restaurants; OR 2.8 (95% CI 1.1–7.6); dry cleaner/launderer; OR 3.8 (95% CI 1.2–12.6); and general farm laborer; OR 4.6 (95% CI 1.8–12.0). Based on small numbers, risk tended to increase with duration in dry cleaning. Giving the deficit risk of colon cancer in farmers, and therefore the low number of farmers among colon cancer controls, the excess risks for mixed farming and general farm laborer should be considered with reservation [20].

A search for associations with small-bowel carcinoid for men and women together included 84 cases and 2070 population controls with excess risks for women in wholesale, food and beverages; OR 8.2 (95% CI 1.9–34.9); and for men in manufacture of bodies for motor vehicles; OR 5.1 (95% CI 1.2–22.1); structural metal preparer/erector; OR 4.3 (95% CI 1.2–15.9); and other construction workers; OR 3.1 (95% CI 1.1–8.6) [21].

### Bone sarcoma

The study included 96 bone sarcoma cases and 2632 population/colon cancer controls [13] with excess risks for blacksmith, toolmaker, machine-tool operator, OR 2.14 (95% CI 1.08–4.26); bricklayer, carpenter, other construction worker; OR 2.93 (95% CI 1.55–5.53); and for the subgroup of carpenter, joiner, parquetry worker; OR 4.25 (95% CI 1.71–10.5), and for manufacture of wood, wood and cork products, straw and plaiting industry; OR 3.58 (95% CI 1.70–7.56). No evidence for association with duration of employment. In total, 18 cases reported work with pesticides; OR 1.63 (95% CI 0.77–3.45); with indication of a dose–response pattern, and of a lower risk in persons using protective equipment; OR 1.36 (95% CI 0.38–4.84).

### Uveal melanoma

Persons with light skin or blue/gray eyes have increased risk of uveal melanoma; corroborated in the French part of the study with OR 2.3 (95% 1.1–4.7) and OR 3.0 (95% CI 1.4–6.3), respectively [22]. Associations previously reported in the literature between occupation and uveal melanoma were confirmed for cooks; OR 2.40 (95% CI 1.35–4.28); welders and sheet metal workers; OR 1.95 (95% CI 1.08–3.52); and service workers not otherwise specified; OR 1.43 (95% CI 1.02–2.00) [12]. In addition, an excess risk was found for launderers, dry-cleaners and pressers; OR 3.14 (95% CI 1.44–6.86). The International Agency for Research on Cancer (IARC) classified welding as a group 1 carcinogen based on the excess risk of ocular melanoma reported in, amongst others, the Rare Cancer Study [23].

The Rare Cancer data were included in a meta-analysis identifying both welding; OR 2.05 (95% CI 1.20–3.51); and occupational cooking; OR 1.81 (95% CI 1.31–2.46) as risk factors, while the increase was marginal only for occupational sunlight exposure; OR 1.37 (95% CI 0.96–1.96) [24]. So, while ultraviolet exposure from sunlight is the most important risk factor for skin melanoma this is not the case for uveal melanoma. As stated by Logan et al. [25] this is consistent with the properties of the adult crystalline lens and cornea to filter out wavelengths below 400 nm. However, short wave light at 400–500 nm, blue light, can reach the posterior uveal tract. Logan et al. noted that arc welding produces short-wave light. It can be added that blue light is emitted also by gas burners often used for professional cooking.

### Thymoma

Due to their rarity and heterogeneous histology, hardly anything is known about risks for thymoma. The Rare Cancer Study included 103 histologically confirmed cases showing a dose–response relationship for tobacco smoking, OR 2.1 (95% CI 1.1–3.9) for > 41 pack-years; and no overall association with alcohol intake, but OR 2.4 (95% CI 1.1–5.4) for > 25 g/day of spirits [26].

### Rare Cancer data in meta-analysis

At the time of the Rare Cancer Study, a 7% increase in breast cancer risk in women per increment of 10 g alcohol/day had been demonstrated [27], but data for men were mixed. Alcohol consumption is high in countries included in the Rare Cancer Study, and the risk of male breast cancer increased with alcohol consumption, being more than fivefold for 9 + drinks/day compared with < 1.5 drinks/day; OR 5.62 (95% 1.54–20.52), [28], Table 3.

**Table 3:** Association between alcohol consumption (g/day) and male breast cancer

EU Rare Cancer Study (22)			NCI Meta-analysis (23)						
Alcohol within last 5 years g/d			Alcohol mostly past year g/d	All studies		Case–control studies		Cohort studies	
	OR	95% CI		OR	95% CI	OR	95% CI	OR	95% CI
0–15	1		0	1		1		1	
			> 0- < 5.93	0.94	0.76–1.16	0.95	0.71–1.27	0.93	0.68–1.27
> 15–30	0.87	0.30–2.47	> 5.73- < 21.65	0.91	0.74–1.13	0.94	0.70–1.25	0.89	0.65–1.21
			> 21.65	1.09	0.88–1.34	1.02	0.77–1.36	1.17	0.85–1.60
> 30–45	1.37	0.46–4.08							
> 45–60	2.28	0.73–7.11	> 45	1.21	0.97–1.53	1.18	0.89–1.56	1.28	0.87–1.90
> 60–75	4.45	1.12–17.66	> 60	1.36	1.04–1.77	1.40	1.02–1.92	1.27	0.79–2.05
> 75–90	4.68	1.07–20.55							
> 90	5.62	1.54–20.52	> 90	1.08	0.74–1.58	1.08	0.68–1.72	1.07	0.55–2.10

EU: European Union; NCI: National Cancer Institute, United States; g/d: gram per day; OR: odds ratio; CI: confidence interval

The Rare Cancer data were included in a meta-analysis of 14 studies where no association was found between alcohol intake and male breast cancer [29], Table 3. The pooled estimate for an intake of 9 + drinks/day compared with non-drinkers was OR 1.08 (95% CI 0.74–1.58). The difference between patterns in the Rare Cancer Study and in the meta-analysis was surprising, but most studies in the meta-analysis reported alcohol consumption during the last year, and misclassification may occur if former drinkers are then classified as non-drinkers. The consistency of the dose–response pattern in the Rare Cancer data makes it difficult to discard the result as a random finding.

### Testing of new findings

The cluster of cholangiocarcinoma cases, both intra-and extrahepatic, from a printing company in Japan [5], was followed up in the Nordic Occupational Cancer Study (NOCCA) [30]. Male workers in printing and related industries had a standardized incidence ratio (SIR) of 2.34 (95% CI 1.45–3.57) for intrahepatic cholangiocarcinoma; and female workers a SIR of 1.95 (95% CI 0.84–3.85), while no association was found for extrahepatic cholangiocarcinoma, ampulla of Vater, and gall bladder. In the Rare Cancer dataset, including gallbladder and extrahepatic cholangiocarcinoma, printing workers had an OR of 2.42 (95% CI 0.81–7.24); being OR 5.78 (95% CI 1.43–23.29) for typesetters [31].

From the majority of Japanese cases both detailed clinical findings [32], and pre-disease levels of liver enzymes measured in blood samples collected at annual health examinations [33], supported a causal association between exposure to 1,2-dichloropropane and cholangiocarcinoma. In 2014, IARC classified 1,2-dichloropropane as carcinogenic to humans (Group 1) based on sufficient evidence in humans that exposure to 1,2-dichloropropane causes cholangiocarcinoma. Dichloromethane was classified as probably carcinogenic to humans (Group 2A) [34].

## Discussion

The Rare Cancer Study showed that well established medical risk factors for the studied diseases could be reproduced, supporting a high validity of the collected data. The study illustrated also how use of cancer controls could lead to spurious findings when the control disease itself was associated with the studied risk factor, as for colon cancer and outdoor work. Results from the Rare Cancer Study provided evidence for classification of welding and 1,2-dichloropropane as carcinogenic to humans. The experiences from the Rare Cancer Study did, however, also illustrate some of the limitations with this study approach.

### Analysis by occupation

Despite major efforts in several countries with identification of 1457 patients, only 1053 of these patients had both the diagnosis confirmed at the pathology review and completed the interview. This meant that only about 100 cases per cancer site could be included in the analysis. Broad occupational categories were therefore used in the analysis, i.e. “wholesale, food and beverages”, and with the expected heterogeneous working tasks, a possible association between a given exposure and a disease would be diluted, with only very strong associations remaining visible. This may explain why the observed ORs rarely exceeded 2–3 with the lower confidence limit close to one.

To overcome the heterogeneity, the analysis could proceed from broad to specific groups. An example was an OR of 2.93 (95% CI 1.55–5.53) for bone sarcomas in “bricklayer, carpenter, other construction worker”, where the excess derived from “carpenter, joiner, parquetry worker”; OR 4.25 (95% CI 1.71–10.5). The possibility of a causal association was strengthened by an increased risk in manufacture of wood, wood and cork products, straw and plaiting industry; OR 3.58 (95% CI 1.70–7.56). A next logical step would have been to collect exposure and clinical data for the carpenters with bone sarcomas, but the group included only six patients, and an attempt to collect detailed data from several countries could easily fail for confidentiality and/or practical reasons.

### Analysis by exposure

Analysis by exposure is a way to get around problems with analysis by occupation. In the study of male biliary tract cancer, an index of exposure to specific chemicals was constructed. The questionnaire job task data were, however, not detailed enough to assess probability, intensity, and duration of exposure, and approximations were needed. It is on this basis not possible to know whether the modest OR of 1.7 (95% CI 1.1–2.8) for exposure to endocrine-disrupting compounds reflected a true value or a deflated value due to lack of sensitivity [15].

A French JEM was used in the male breast cancer study to aggregate persons from occupations exposed to organic solvents; indicating an association with trichloroethylene; high exposure OR 1.9 (95% CI 1.1–3.3) [18]. Again, lack of details in the questionnaires might limit correct allocation, as study subjects had worked in eight countries over a period of three to four decades. NOCCA-data on male breast cancer were combined with a JEM for Nordic countries, showing for trichloroethylene an OR of 1.55 (95% CI 0.64–3.76) [35]. These two explorative studies indicate that the association between exposure to trichloroethylene and male breast cancer deserves further scrutiny. The NOCCA-data showed a reduced risk of male breast cancer for men with physical workloads; OR 0.78 (95% CI 0.67–0.91) [35]. This was not confirmed in the Rare Cancer Study with an OR of 0.9 (95% CI 0.6–1.4) for agriculture, and OR 2.4 (95% CI 1.0–5.6) for forestry/logging [17]; both physically demanding industries.

### Reflections

It was an underlying assumption of the Rare Cancer Study that rare cancers do not occur at random but result from rare exposures and/or rare susceptibility to exposures, and that these risks could be identified in a systematic search for associations in a large dataset.

The study demonstrated, however, that the approach had build-in limitations. For each rare cancer site, systematic tabulation across occupations revealed some increased ORs, but mostly in the order of 2–3. These ORs may represent etiological associations buried in noise, or they may simply reflect random variation in tabulation of many associations. There is no way to solve this question within the dataset itself. The lack of sensitivity of the Rare Cancer Study for detection of signals is a characteristic shared with other explorative studies. An example is the NOCCA-study, where Nordic census data were linked individually with cancer data for 15 million persons [36], and where the largest divergences between occupations were for cancers associated with tobacco and alcohol.

It is characteristic for reports where alert clinicians provided hints on occupational risks for rare cancers that they had very detailed data on the patients; both on exposures and histology, and sometimes even on pre-diagnostic biomarkers. In comparison, data in explorative studies are very limited. The most constructive use of explorative studies in their present form is therefore for identification of consistent findings across studies, as for uveal melanoma in welders and cooks, and for targeted studies of already suspected associations, as for cholangiocarcinoma in printers. It is therefore important to document and store the data, and to make them easily available for researchers.

As stated in the preamble to the IARC Monographs on the identification of carcinogenic hazards to humans [37], evidence for a causal association in human studies is strengthened by consistent findings. Outcomes from explorative studies may play an important role here, and as illustrated above data from the Rare Cancer Study have proved valuable in this context. Explorative studies may also form part of surveillance systems for occupational safety and health [38]. The usefulness of explorative studies in identifying new possible forms of work risks to better protect workers could be further enhanced, if risks revealed in the statistical analysis could be followed up by confirmatory studies of the relevant sub-groups with individual data on diagnoses, exposures, and possible confounders. However, the possibility for such targeted enrichment of explorative studies is limited by data protection rules.

The present disease pattern reflects the history of our life, and as the working conditions have changed over time one could question the relevance of results from explorative studies for future protection of workers. Some aspects of working environments are, however, relatively stable over time, and there is no doubt that the identification of carcinogenic compounds and work processes has in itself been a driver for changes in working conditions [39].

In conclusion, the Rare Cancer Study proved it possible to collect valid data with interviews conducted in several languages. However, despite efforts across Europe only about 100 cases per cancer site could be identified, confirmed, and interviewed within the study period. The sensitivity of explorative studies in the search for etiological leads is limited by use of broad occupational groups and lack of access to individual, detailed exposure and clinical data. The Rare Cancer data set is a valuable source for comparisons of findings across explorative studies and for targeted confirmatory studies.

## Publications from the European multi-center case–control study on rare cancers of unknown etiology

### Male breast cancer

Occupational exposure to organic solvents and risk of male breast cancer: a European multicenter case–control study.

Laouali N, Pilorget C, Cyr D, Neri M, Kaerlev L, Sabroe S, Gorini G, Richiardi L, Morales-Suárez-Varela M, Llopis-Gonzalez A, Ahrens W, Jöckel KH, Afonso N, Eriksson M, Merletti E, Olsen J, Lynge E, Guénel P.

Scand J Work Environ Health. 2018 May 1;44(3):310–322. https://doi.org/10.5271/sjweh.3717. Epub 2018 Feb 6.

Occupation and occupational exposure to endocrine disrupting chemicals in male breast cancer: a case–control study in Europe.

Villeneuve S, Cyr D, Lynge E, Orsi L, Sabroe S, Merletti F, Gorini G, Morales-Suarez-Varela M, Ahrens W, Baumgardt-Elms C, Kaerlev L, Eriksson M, Hardell L, Févotte J, Guénel P.

Occup Environ Med. 2010 Dec;67(12):837–44. https://doi.org/10.1136/oem.2009.052175. Epub 2010 Aug 25.

Breast cancer in priests: follow-up of an observation made 167 years ago.

Fritschi L, Guenel P, Ahrens W; European Study Group on Occupational Causes of Rare Cancers.

Eur J Epidemiol. 2010 Mar;25(3):219–21. https://doi.org/10.1007/s10654-010-9426-8. Epub 2010 Jan 21. No abstract available.

Alcohol drinking may increase risk of breast cancer in men: a European population-based case–control study.

Guénel P, Cyr D, Sabroe S, Lynge E, Merletti F, Ahrens W, Baumgardt-Elms C, Ménégoz F, Olsson H, Paulsen S, Simonato L, Wingren G.

Cancer Causes Control. 2004 Aug;15(6):571–80.

### Male breast cancer, international collaboration

Tobacco and alcohol in relation to male breast cancer: an analysis of the male breast cancer pooling project consortium.

Cook MB, Guénel P, Gapstur SM, van den Brandt PA, Michels KB, Casagrande JT, Cooke R, Van Den Eeden SK, Ewertz M, Falk RT, Gaudet MM, Gkiokas G, Habel LA, Hsing AW, Johnson K, Kolonel LN, La Vecchia C, Lynge E, Lubin JH, McCormack VA, Negri E, Olsson H, Parisi D, Petridou ET, Riboli E, Sesso HD, Swerdlow A, Thomas DB, Willett WC, Brinton LA.

Cancer Epidemiol Biomarkers Prev. 2015 Mar;24(3):520–31. https://doi.org/10.1158/1055-9965.EPI-14-1009. Epub 2014 Dec 16.

Anthropometric and hormonal risk factors for male breast cancer: male breast cancer pooling project results.

Brinton LA, Cook MB, McCormack V, Johnson KC, Olsson H, Casagrande JT, Cooke R, Falk RT, Gapstur SM, Gaudet MM, Gaziano JM, Gkiokas G, Guénel P, Henderson BE, Hollenbeck A, Hsing AW, Kolonel LN, Isaacs C, Lubin JH, Michels KB, Negri E, Parisi D, Petridou ET, Pike MC, Riboli E, Sesso HD, Snyder K, Swerdlow AJ; European Rare Cancer Study Group, Trichopoulos D, Ursin G, van den Brandt PA, Van Den Eeden SK, Weiderpass E, Willett WC, Ewertz M, Thomas DB.

J Natl Cancer Inst. 2014 Mar;106(3):djt465. https://doi.org/10.1093/jnci/djt465. Epub 2014 Feb 19. Erratum in: J Natl Cancer Inst. 2014 May;106(5):dju117.

### Biliary tract carcinoma

**Biliary tract** cancer in male printers and typesetters in the European rare cancer case–control study.

Ahrens W, Merletti F, Mirabelli D.

Occup Environ Med. 2014 Aug;71(8):591–2. https://doi.org/10.1136/oemed-2014-102322. Epub 2014 Jun 10. No abstract available.

Occupational exposure to pesticides and bile tract carcinoma in men: results from a European multicenter case–control study.

Schmeisser N, Kaerlev L, Bourdon-Raverdy N, Ganry O, Llopis-González A, Guénel P, Hardell L, Merletti F, Zambon P, Morales-Suárez-Varela M, Olsen J, Olsson H, Vyberg M, Ahrens W.

Cancer Causes Control. 2010 Sep;21(9):1493–502. https://doi.org/10.1007/s10552-010-9578-6. Epub 2010 Jun 9.

Occupational exposure to endocrine-disrupting compounds and biliary tract cancer among men.

Ahrens W, Mambetova C, Bourdon-Raverdy N, Llopis-González A, Guénel P, Hardell L, Merletti F, Morales-Suárez-Varela M, Olsen J, Olsson H, Vyberg M, Zambon P.

Scand J Work Environ Health. 2007 Oct;33(5):387–96. Erratum in: Scand J Work Environ Health. 2008 Jun;34(3):234.

Risk factors for extrahepatic biliary tract carcinoma in men: medical conditions and lifestyle: results from a European multicentre case–control study.

Ahrens W, Timmer A, Vyberg M, Fletcher T, Guénel P, Merler E, Merletti F, Morales M, Olsson H, Olsen J, Hardell L, Kaerlev L, Raverdy N, Lynge E.

Eur J Gastroenterol Hepatol. 2007 Aug;19(8):623–30.

### Uveal melanoma

Occupational exposure to endocrine-disrupting chemicals and the risk of uveal melanoma.

Behrens T, Lynge E, Cree I, Lutz JM, Eriksson M, Guénel P, Merletti F, Morales-Suarez-Varela M, Afonso N, Stengrevics A, Stang A, Févotte J, Sabroe S, Llopis-González A, Gorini G, Hardell L, Ahrens W.

Scand J Work Environ Health. 2012 Sep;38(5):476–83. https://doi.org/10.5271/sjweh.3265. Epub 2011 Dec 17.

Pesticide exposure in farming and forestry and the risk of uveal melanoma.

Behrens T, Lynge E, Cree I, Lutz JM, Eriksson M, Guénel P, Merletti F, Morales-Suarez-Varela M, Afonso N, Stengrevics A, Févotte J, Sabroe S, Llopis-González A, Gorini G, Hardell L, Stang A, Ahrens W.

Cancer Causes Control. 2012 Jan;23(1):141–51. https://doi.org/10.1007/s10552-011-9863-z. Epub 2011 Nov 4.

Occupational exposure to electromagnetic fields and sex-differential risk of uveal melanoma.

Behrens T, Lynge E, Cree I, Sabroe S, Lutz JM, Afonso N, Eriksson M, Guénel P, Merletti F, Morales-Suarez-Varela M, Stengrevics A, Févotte J, Llopis-González A, Gorini G, Sharkova G, Hardell L, Ahrens W.

Occup Environ Med. 2010 Nov;67(11):751–9. https://doi.org/10.1136/oem.2009.052225. Epub 2010 Aug 25.

Hormonal exposures and the risk of uveal melanoma.

Behrens T, Kaerlev L, Cree I, Lutz JM, Afonso N, Eriksson M, Guénel P, Merletti F, Morales-Suarez-Varela M, Stengrevics A, Sabroe S, Cyr D, Llopis-González A, Gorini G, Sharkova G, Hardell L, Ahrens W.

Cancer Causes Control. 2010 Oct;21(10):1625–34. https://doi.org/10.1007/s10552-010-9591-9. Epub 2010 Jun 4.

Occupational risks for uveal melanoma results from a case–control study in nine European countries.

Lutz JM, Cree I, Sabroe S, Kvist TK, Clausen LB, Afonso N, Ahrens W, Ballard TJ, Bell J, Cyr D, Eriksson M, Févotte J, Guénel P, Hardell L, Jöckel KH, Miranda A, Merletti F, Morales-Suarez-Varela MM, Stengrevics A, Lynge E.

Cancer Causes Control. 2005 May;16(4):437–47.

### Uveal melanoma, French data only

Occupational risk factors, ultraviolet radiation, and ocular melanoma: a case–control study in France.

Guénel P, Laforest L, Cyr D, Févotte J, Sabroe S, Ufour C, Lutz JM, Lynge E.

Cancer Causes Control. 2001 Jun;12(5):451–9.

### Bone sarcoma

Occupational factors and risk of adult bone sarcomas: a multicentric case–control study in Europe.

Merletti F, Richiardi L, Bertoni F, Ahrens W, Buemi A, Costa-Santos C, Eriksson M, Guénel P, Kaerlev L, Jöckel KH, Llopis-Gonzalez A, Merler E, Miranda A, Morales-Suárez-Varela MM, Olsson H, Fletcher T, Olsen J.

Int J Cancer. 2006 Feb 1;118(3):721–7.

### Thymoma

Tobacco smoking and alcohol consumption as risk factors for **thymoma**—A European case–control study.

**Eriksson** M, **Kaerlev** L, Johansen P, Afonso N, Ahrens W, Costa-Pereira A, Guénel P, Jöckel KH, Gonzalez AL, Merletti F, Suárez-Varela MM, Trétarre B, Wingren G, Richiardi L, Sabroe S.

Cancer Epidemiol. 2019 Aug;61:133–138. https://doi.org/10.1016/j.canep.2019.06.008. Epub 2019 Jun 26.

### Mycosis fungoides

Occupational exposure to chlorinated and petroleum solvents and mycosis fungoides.

Morales-Suárez-Varela MM, Olsen J, Villeneuve S, Johansen P, Kaerlev L, Llopis-González A, Wingren G, Hardell L, Ahrens W, Stang A, Merletti F, Gorini G, Aurrekoetxea JJ, Févotte J, Cyr D, Guénel P.

J Occup Environ Med. 2013 Aug;55(8):924–31. https://doi.org/10.1097/JOM.0b013e3182941a1c.

Occupational sun exposure and mycosis fungoides: a European multicenter case–control study.

Morales-Suárez-Varela MM, Olsen J, Johansen P, Kaerlev L, Guénel P, Arveux P, Wingren G, Hardell L, Ahrens W, Stang A, Llopis A, Merletti F, Guillen-Grima F, Masala G.

J Occup Environ Med. 2006 Apr;48(4):390–3.

Occupational exposures and mycosis fungoides. A European multicentre case–control study (Europe).

Morales-Suárez-Varela MM, Olsen J, Johansen P, Kaerlev L, Guénel P, Arveux P, Wingren G, Hardell L, Ahrens W, Stang A, Llopis A, Merletti F, Aurrekoetxea JJ, Masala G.

Cancer Causes Control. 2005 Dec;16(10):1253–9.

Occupational risk factors for mycosis fungoides: a European multicenter case–control study.

Morales-Suárez-Varela MM, Olsen J, Johansen P, Kaerlev L, Guénel P, Arveux P, Wingren G, Hardell L, Ahrens W, Stang A, Llopis A, Merletti F, Aurrekoetxea JJ, Masala G.

J Occup Environ Med. 2004 Mar;46(3):205–11.

Viral infection, atopy and mycosis fungoides: a European multicentre case–control study.

Morales MM, Olsen J, Johansen P, Kaerlev L, Guénel P, Arveux P, Wingren G, Hardell L, Ahrens W, Stang A, Llopis A, Merletti F, Villanueva MA.

Eur J Cancer. 2003 Mar;39(4):511–6.

Are alcohol intake and smoking associated with mycosis fungoides? A European multicentre case–control study.

Morales Suárez-Varela MM, Olsen J, Kaerlev L, Guénel P, Arveux P, Wingren G, Hardell L, Ahrens W, Stang A, Llopis-Gonzalez A, Merletti F, Guillén-Grima F, Johansen P.

Eur J Cancer. 2001 Feb;37(3):392–7.

### Small bowel adenocarcinoma and carcinoids

Glutathione S-transferase genotype and p53 mutations in adenocarcinoma of the small intestine.

Nørum Pedersen L, Kaerlev L, Stubbe Teglbjaerg P, Olsen J, Eriksson M, Guenel P, Ahrens W, Ballard T, Autrup H.

Scand J Gastroenterol. 2003 Aug;38(8):845–9.

Occupational risk factors for small bowel carcinoid tumor: a European population-based case–control study.

Kaerlev L, Teglbjaerg PS, Sabroe S, Kolstad HA, Ahrens W, Eriksson M, Guénel P, Hardell L, Cyr D, Ballard T, Zambon P, Morales Suárez-Varela MM, Stang A, Olsen J.

J Occup Environ Med. 2002 Jun;44(6):516–22.

The importance of smoking and medical history for development of small bowel carcinoid tumor: a European population-based case–control study.

Kaerlev L, Teglbjaerg PS, Sabroe S, Kolstad HA, Ahrens W, Eriksson M, Guénel P, Gorini G, Hardell L, Cyr D, Zambon P, Stang A, Olsen J.

Cancer Causes Control. 2002 Feb;13(1):27–34.

Medical risk factors for small-bowel adenocarcinoma with focus on Crohn disease: a European population-based case–control study.

Kaerlev L, Teglbjaerg PS, Sabroe S, Kolstad HA, Ahrens W, Eriksson M, Guénel P, Hardell L, Launoy G, Merler E, Merletti F, Stang A.

Scand J Gastroenterol. 2001 Jun;36(6):641–6.

Is there an association between alcohol intake or smoking and small bowel adenocarcinoma? Results from a European multi-center case–control study.

Kaerlev L, Teglbjaerg PS, Sabroe S, Kolstad HA, Ahrens W, Eriksson M, Guénel P, Hardell L, Launoy G, Merler E, Merletti F, Stang A, Olsen J.

Cancer Causes Control. 2000 Oct;11(9):791–7.

Occupation and small bowel adenocarcinoma: a European case–control study.

Kaerlev L, Teglbjaerg PS, Sabroe S, Kolstad HA, Ahrens W, Eriksson M, González AL, Guénel P, Hardell L, Launoy G, Merler E, Merletti F, Suárez-Varela MM, Stang A.

Occup Environ Med. 2000 Nov;57(11):760–6.

### Methodology

European multi-centre case–control study on risk factors for rare cancers of unknown aetiology.

Lynge E, Afonso N, Kaerlev L, Olsen J, Sabroe S, Ahrens W, Eriksson M, Guénel P, Merletti F, Stengrevics A, Suarez-Varela M, Costa-Pererra A, Vyberg M.

Eur J Cancer. 2005 Mar;41(4):601–12. Epub 2005 Jan 26.

Reliability of data from next-of-kin: results from a case–control study of occupational and lifestyle risk factors for cancer.

Kaerlev L, Lynge E, Sabroe S, Olsen J.

Am J Ind Med. 2003 Sep;44(3):298–303.

Colon cancer controls versus population controls in case–control studies of occupational risk factors.

Kaerlev L, Lynge E, Sabroe S, Olsen J.

BMC Cancer. 2004 Apr 22;4:15.
